# Health system requirements for hearing care services

**DOI:** 10.2471/BLT.19.243683

**Published:** 2019-10-01

**Authors:** Shelly Chadha, Kaloyan Kamenov, Alarcos Cieza

**Affiliations:** a Department of noncommunicable diseases, World Health Organization, avenue Appia 20, 1211 Geneva 27, Switzerland.

Hearing loss is a public health challenge. The World Health Organization (WHO) estimates that 466 million people experience moderate or higher levels of hearing loss and that this number could double by 2050 if the current demographic trends continue.^1^ More than a billion young people are at risk of hearing loss due to listening to loud sounds alone, reflecting the magnitude of this problem.^2^ Hearing loss is the fifth leading cause for years lived with disability worldwide,^3^ mainly due to its impact on interpersonal communication, cognition, education, employment and social participation.^4^^–^^6^ Moreover, unaddressed hearing loss poses a huge global financial burden, costing over 750 billion United States dollars annually.^7^

Fortunately, many of the causes that lead to hearing loss can be avoided through preventative public health actions. Those who experience hearing loss can benefit from early identification and intervention. Hearing aids, cochlear implants, use of sign language and assistive technologies are some of the available options to ensure that no one lives with unaddressed hearing loss. The 2017 World Health Assembly Resolution *Prevention of deafness and hearing loss*^1^ calls on Member States across all regions for stronger action in this field. This resolution has led several Member States to strengthen their ear and hearing care services while others are starting to develop public health strategies on hearing loss.^8^ However, challenges including lack of trained workforce, inadequate financial resources and low awareness still need to be met.

These challenges can be tackled through the integration of ear and hearing care into health systems as part of universal health coverage (UHC). In 2015, when the United Nations General Assembly adopted the sustainable development goals, WHO and its Member States committed to achieve UHC by 2030.^9^ This target highlights the importance of primary health care and health system strengthening as the core approaches for delivering comprehensive person-centred care without causing financial hardship. Integrating ear and hearing care within these public health approaches is the key to ensuring sustainable and equitable access to services for everyone. In practice, this integration would require implementing three strategies. First, including cost–effective interventions for ear and hearing care into mainstream health services. Second, training community health workers to raise awareness for hearing loss prevention and identification at the community level.^10^^,^^11^ This step could mitigate the stigma associated with this condition and ensure that people are able to access care for hearing loss and the common ear diseases that often lead to it. Third, developing effective policies for preventative measures like noise reduction and rational drug use. Fourth, ensuring access to sign-language education^6^ and hearing devices.^12^Over the past few years, WHO and partners have provided support to Member States in ear and hearing care.^8^ The World Hearing Day has contributed to raising awareness on the prevention, identification and management of hearing loss, both at level of policy-makers and the public. WHO has developed evidence-based recommendations to reduce the risks of hearing loss and created tools to support governments in planning and implementing ear and hearing care services.

This theme issue draws further attention towards this topic, highlighting the impact of unaddressed hearing loss^4^^–^^6^ and the inequities in ear and hearing care services.^13^^,^^14^ This issue also suggests potential solutions that seek to integrate ear and hearing care within the overall spectrum of services provided through health systems, and focuses on building capacity of health-care providers in ear and hearing care.^10^^,^^11^ Papers present technological solutions that can improve access to hearing care and facilitate its integration into health systems^15^^,^^16^ and provide examples of innovative policies that could serve as models for improving access to services in under-served areas of the world.^6^^,^^12^

The theme issue sets the stage for the First Membership Assembly of the World Hearing Forum, which will take place this December at WHO headquarters in Geneva, Switzerland.^17^ This meeting will bring together all stakeholders in this field, to strengthen collaborations for promoting hearing care globally. This issue is also a precursor to the *World report on hearing*, which will recommend a package of evidence-based interventions for improving ear and hearing care through health system strengthening.
